# Elevated iron levels in tears of patients diagnosed with *WDR45* X-linked optic atrophy

**DOI:** 10.1186/s13023-026-04315-4

**Published:** 2026-04-06

**Authors:** Marina Michelson, Alon Zahavi, Tal Zobok, Keren Yosovich, Lubov Blumkin, Idit Maharshak, Dorit Lev, Olga Girshevitz, Nitza Goldenberg-Cohen

**Affiliations:** 1 Rina Mor Institute of Medical Genetics, Wolfson Medical Center, Holon, 5822012 Israel; 2 Faculty of Medicine, Tel Aviv University, Tel Aviv, 6997801 Israel; 3 Genetic Institute of Maccabi Healthcare Services, Holon, Israel; 4 Depatment of Ophthalmology and Laboratory of Eye Research, Felsenstein Medical Research Center, Rabin Medical Center, Petach Tikva, 4941492 Israel; 5 Faculty of Medicine, Hebrew University Hadassah Medical School, Jerusalem, 91120 Israel; 6 Magen Center for Rare Diseases, Wolfson Medical Center, Holon, 5822012 Israel; 7 Department of Ophthalmology, Wolfson Medical Center, Holon, 5822012 Israel; 8 Faculty of Engineering and Institute of Nanotechnology and Advanced Materials, Bar-Ilan University, Ramat Gan, 5290002 Israel; 9 Department of Ophthalmology, Bnai-Zion Medical Center, Haifa, 3339419 Israel; 10 Krieger Eye Research Laboratory, Ruth and Bruce Faculty of Medicine, Technion -- Institute of Technology, Haifa, 3200003 Israel

**Keywords:** *WDR45*, Iron accumulation, X-link optic atrophy, PIXE, particle-induced X-ray emission, Tears

## Abstract

**Background:**

Pathogenic variants in *WDR45* are associated with iron deposits in the basal ganglia. We aimed to determine iron levels in tears of patients with *WDR45* X-linked optic atrophy compared to healthy controls.

**Methods:**

The study group included two brothers aged 26 and 32 years and a 3-year-old girl from another family diagnosed with optic atrophy, all of whom harbored a novel pathogenic variant in *WDR45*. Tear samples were collected from the inferior fornix using Schirmer filter strips and analyzed for trace elements using a particle-induced X-ray emission (PIXE) technique and a Fast X123 SDD70 (C2) detector. Concentrations were calculated as the difference from unused Schirmer strips. Findings were compared with 28 healthy subjects, 21 females and 7 males, aged 18–50 years (mean 28.5 ± 9.09 years).

**Results:**

All three patients with *WDR45* X-linked optic atrophy exhibited elevated iron levels in their tears compared to healthy controls. No differences from the control group were detected for other trace elements.

**Conclusions:**

This is the first study to show higher-than-normal iron levels in the tears of patients with X-linked optic atrophy due to a variant in *WDR45.* The findings broaden our understanding of the role of the *WDR45* gene in disorders related to abnormal iron pathophysiology. PIXE analysis is a highly sensitive technique for measuring trace elements in tears. Further clinical studies are needed to investigate potentially novel genotype-phenotype correlations. The application of these findings in clinical practice may benefit patient care.

## Background

The *WDR45* gene is located on the X chromosome and is ubiquitously expressed in all tissues [1]. It encodes a member of the WD repeat protein domain, phosphoinositide-interacting protein 4 (WIPI-4), which plays a pivotal role in numerous cellular processes, including the cell cycle, signal transduction, and apoptosis. Alternative splicing in the *WDR45* gene results in multiple transcript variants that lack tissue specificity and do not have a defined role in specific functions.

Pathogenic variants in *WDR45* are associated with a broad phenotypic spectrum including static cognitive impairment, intellectual disability, motor impairment, speech difficulties, and epilepsy [1-5]. The clinical continuum comprises six distinct albeit interrelated disorders: beta-propeller protein-associated neurodegeneration (BPAN), Rett-like syndrome, intellectual disability, developmental and epileptic encephalopathy, early-onset epileptic encephalopathy, and West syndrome [6]. Most of the reported patients are female with *de novo* heterozygous variants. There are wide phenotypic variations in both genders, with no substantiation of a genotype-phenotype correlation [7]. Studies have reported iron deposits in the basal ganglia in patients with *WDR45*-associated disorders, suggesting a disruption in iron metabolism as a contributing pathogenic factor [7, 8].

Optic atrophy is characterized clinically by progressive loss of retinal ganglion cells and axonal degeneration, leading to visual impairment and blindness [9]. It may result from a diverse array of both acquired and inherited factors. The optic nerve is highly sensitive to oxidative stress, and any impairment in oxygenation can lead to visual loss. Inherited optic atrophy is a highly heterogeneous group of disorders that can manifest either as an isolated condition or as part of a syndrome [10]. Both forms may exhibit various modes of inheritance, including autosomal dominant, recessive, X-linked, and mitochondrial. Common mechanisms underlying optic atrophy involve mitochondrial dysfunction, namely disturbances in mitochondrial dynamics, alterations in the oxidative phosphorylation network, and interplay between the endoplasmic reticulum and mitochondria [11], all of which lead to neurodegeneration [12]. Bilateral optic atrophy has been described in a patient with a BPAN phenotype and sensorineural hearing loss [13].

A recent study reported, for the first time, two families with optic atrophy caused by pathogenic variants in *WDR45* [14]. *WDR45* lies within the OPA2 locus associated with X-linked optic atrophy, and classical *WDR45*-related disorders are characterized by iron accumulation in the basal ganglia. Based on the known association between WDR45 dysfunction and iron accumulation, our aim was to explore whether iron dysregulation might also contribute to optic nerve atrophy in this context. Although no direct biological pathway between tear iron and optic nerve pathology has been established, we examined whether tear iron levels might reflect early or localized dysregulation related to WDR45.

## Methods

The study protocol and consent procedures conformed to the tenets of the Declaration of Helsinki and local ethical guidelines. The study protocol was approved by the local Institutional Review Board. Written informed consent was obtained from the participants prior to enrollment.

The study group included two brothers, aged 32 and 26 years from one family previously described by Gazit et al. [14], who had isolated optic atrophy and a pathogenic variant in *WDR45*, and a 3-year-old girl (patient 3) from another family with optic atrophy and global developmental delay, in whom a different pathogenic *WDR45* variant was identified.

The patients were followed by a multidisciplinary team of clinical geneticists, ophthalmologists, neurologists, and neuroradiologists. All underwent molecular genetic testing and neuroimaging.

The control group included 28 healthy adult volunteers who regularly participated in studio-based fitness activities and were partially described in an earlier publication by our group [15]. Eligibility criteria included age 18–50 years and absence of ocular conditions or behaviors that could affect tear composition. Specifically, individuals were excluded if they used contact lenses, had a diagnosis of glaucoma or were using chronic ophthalmic medications, had dry eye disease or anterior segment pathology, had swum within 2 h prior to sampling, or had recently applied eye-area cosmetics such as eyeliner or mascara. Tear samples were collected from all participants in both groups for elemental analysis.

### Sanger sequencing

Confirmation and further familial segregation of the variant in *WDR45* was performed using direct Sanger sequencing (3500 Genetic Analyzer Applied Biosystems).

### Targeted *WDR45*-RNA sequencing analysis

Targeted next-generation RNA sequencing was performed at MNG Laboratories, Medical Neurogenetics, LLC (Atlanta, GA, USA) using an Illumina platform with TruSeq Stranded Total RNA library preparation kit (Illumina, Inc., San Diego, CA, USA) generated from the patient’s peripheral blood sample (age 26 years). The sequencing reads were aligned with HISAT2 and analyzed with StringTie to quantify transcript ratios and splice site usage [16].

The test samples were compared with a panel of tissue-specific reference samples to calculate Z-scores for the assessment of expression changes and their likely significance. This targeted analysis was intended as an exploratory expression study to assess the splicing effect of the c.236-1G > T variant in peripheral blood.

### Clinical expression studies

A full ophthalmological examination was performed on all three patients, including visual acuity, cycloplegic refraction, slit-lamp biomicroscopy (CSO, Florence, Italy), orthoptic evaluation, optical coherence tomography (OCT; Heidelberg, Franklin, MA, USA), and electroretinography (ERG). Visual field testing (Humphry, Zeiss, Germany) was performed on the two brothers but not the girl because of her young age. Systemic evaluation and neurological examination, in addition to magnetic resonance imaging (MRI), were performed in all patients.

### Tear sampling

The eyes of all study participants were anesthetized with oxybuprocaine hydrochloride 4% drops (Localin) for 1 min. The PIXE analysis was conducted separately for each eye, and the mean value of the two eyes was used for each individual in the comparative analysis. An unused Schirmer strip with and without anesthesia served as a negative control.

### Particle induced X-ray emission (PIXE)

The PIXE analysis was conducted separately for each eye, and the mean value of the two eyes was used for each individual in the comparative analysis. The optimized PIXE technique used for tear analysis was reported previously by our group [17]. In brief, a 2.01 (± 0.01) mega electron Volts (MeV) proton beam was generated by a 1.7 megavolt (MV) tandem Pelletron accelerator (National Electrostatics Corp., Middleton, WI, USA) and collimated to a diameter of 1.5 mm and current of ~ 7 nanoampere (nA). The integrated charge (Q) of 3 microcoulomb (µC) was used. PIXE data were acquired with a Fast X123 Silicon Drift Detector (SDD) 70 (C2) detector (Amptek, Bedford, MA, USA) with a nominal surface area of 30 mm^2^, Si crystal thickness of 500 μm, and minimal thick silicon nitride (Si3N4) window (40 nm). The normal incident beam was used in all measurements. The detector was positioned at 45˚ to the beam normal (IBM geometry), and a funny filter (FF) consisting of 100 mm Kapton film with a 1.5% effective area hole was used for all measurements. At least three measurements were carried out for each sample in different areas. PIXE spectra were processed with the GUPIX package [18]. Measured concentrations were based on three replicates for each sample. The differences in concentrations of trace elements between the samples and plain Schirmer strips were calculated. Findings in the study patients were compared to the healthy control subjects.

### Magnetic resonance imaging

Brain MR images in the two brothers were acquired using a 1.5T Philips Ingenia system (Philips Medical Systems, Netherlands), employing a standardized protocol that included 3D T1-weighted turbo field echo (3D TFE), T2-weighted fast spin echo (FSE), 3D fluid-attenuated inversion recovery (FLAIR), susceptibility-weighted imaging (SWI), and diffusion-weighted echo-planar imaging (DWI).

## Results

### Clinical characterization

#### Study group

Two brothers of Ashkenazi Jewish ancestry were followed at the Magen Center for Rare Diseases by a multidisciplinary team and referred for further evaluation of progressive optic atrophy caused by a hemizygous pathogenic variant, *WDR45*: c.236-1G > T,p.Val80Leu.

**Fig. 1:**
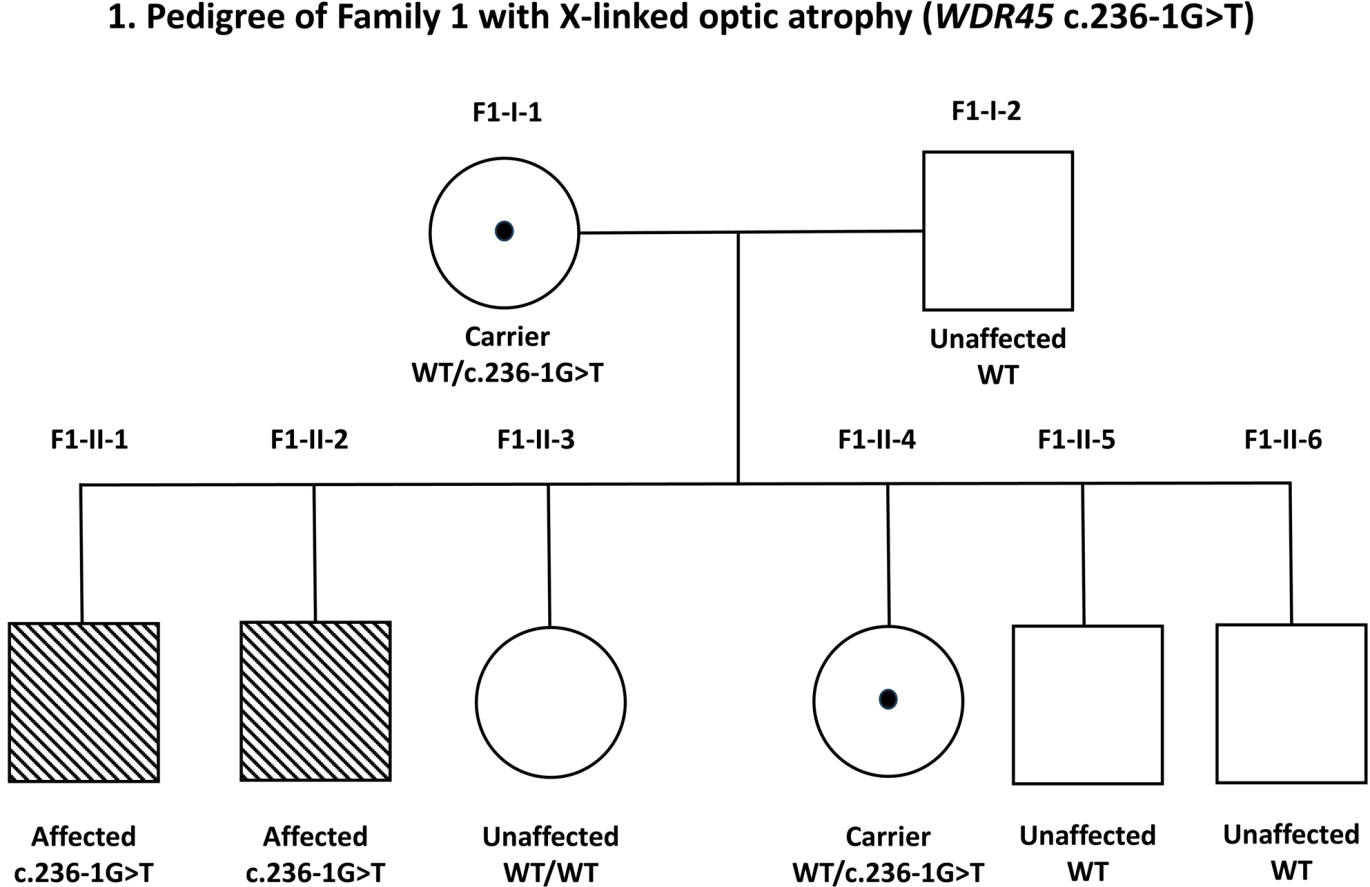
Pedigree of Family 1 with X-linked optic atrophy associated with the *WDR45* splice-site variant c.236-1G > T. The two affected brothers (F1-II-1 and F1-II-2) are hemizygous for the variant. Their mother (F1-I-1) and one sister (F1-II-4) are heterozygous (carriers), whereas the father (F1-I-2) and the remaining siblings carry the wild-type allele and are unaffected. Squares indicate males and circles indicate females. Shaded symbols indicate affected individuals; a central dot indicates heterozygous carriers; open symbols indicate unaffected individuals. Genotypes are shown beneath each symbol. WT, wild type

Their 65-year-old mother and 40-year-old sister were found to be heterozygous for the same variant. The mother had optic disc pallor, and the sister had myopia on clinical examination. There was no family history of neurodegenerative or neurodevelopmental disorders. The pedigree is shown in Fig. 1.

The older brother (patient 1), age 32 years, had an unremarkable perinatal history. Early developmental delay was noted, but he attended a regular school and successfully graduated from high school. At age 20 years, he was diagnosed with obsessive-compulsive behavior and autistic features. There was no history of seizures, and no evidence of regression or deterioration in communication, cognitive, or motor function. He currently lives and works independently.

**Fig. 2:**
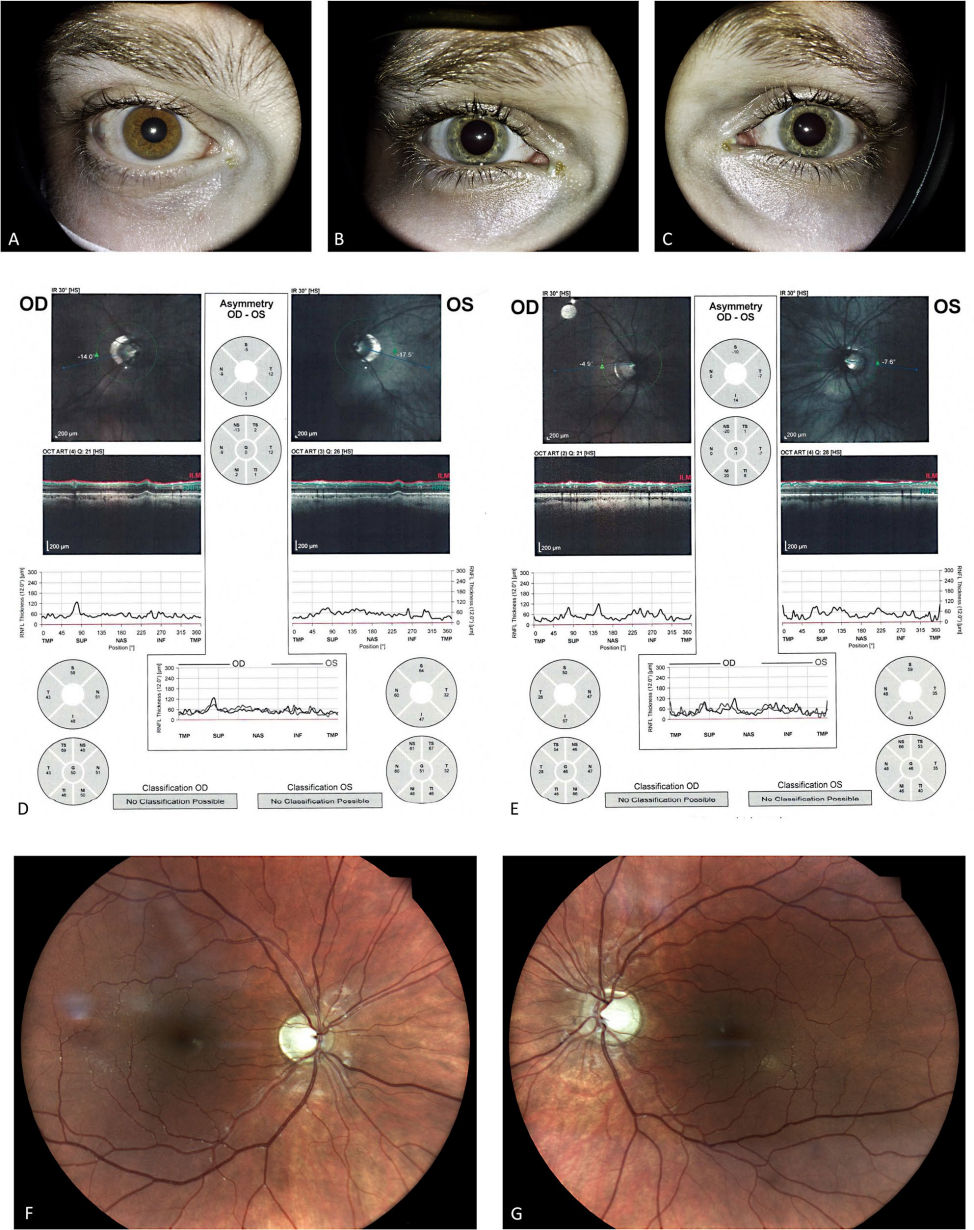
Anterior segment, OCT, and fundus, patients 1 and 2. Anterior segment of the older brother (**A**) and the younger brother (**B**, **C**) shows clear corneas with no evidence of iron deposits. Retinal nerve fiber layer OCT scans show general thinning of the peripapillary nerve fibers. Average thickness measures 50 μm on the right side and 51 μm on the left in the elder brother (**D**) and 46 μm bilaterally in the younger brother (**E**). Fundus colour picture demonstrated prominent optic disc pallor in the right (**F**) and left (**G**) eyes of patient 2, with no macular or retinal pathologies

**Fig. 3:**
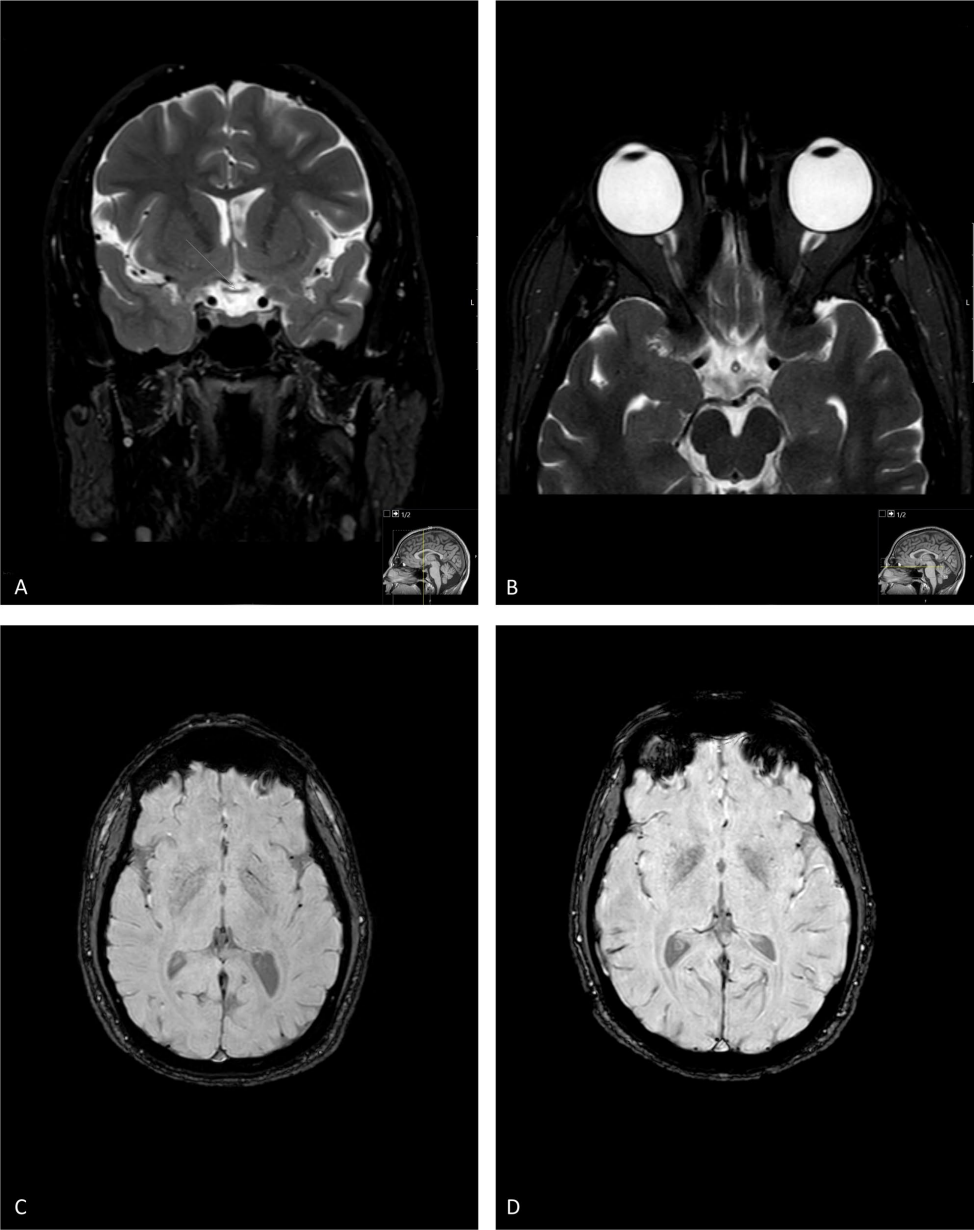
Brain MRI. T2 coronal (**A**) and axial (**B**) scans of patient 1 demonstrate thinning of the optic nerves and chiasm, with no other pathological findings. Susceptibility-weighted imaging (SWI) of Patient 1 (**C**) and Patient 2 (**D**) revealed no abnormal iron accumulation within the basal ganglia

Ocular pathology was noted at age 3.5 years when the patient was evaluated for vision difficulties. The initial diagnosis was progressive myopia. By age 7 years, the myopia worsened, and optic atrophy was detected. Corrected visual acuity at the time of this study was 20/150 in both eyes with myopia 15.0D, normal anterior segment with no evidence of iron deposits (Fig. 2A), and prominent disc pallor. Visual evoked potential testing revealed low amplitude and prolonged latency times, and subsequent OCT demonstrated general thinning of the peripapillary nerve fibers (average thickness, 50 and 51 μm in the right and left eye, respectively) (Fig. 2D). Visual field assessments revealed an extensive temporal defect and residual nasal field. Brain MRI indicated optic nerve atrophy without any other pathological findings (Fig. 3). Symptoms observed on neurological evaluation included abnormal head tilt with chin up, clumsiness related to rapid alternating movements, and synkinesis. Genetic evaluation performed at age 12 years ruled out G11778A, T114484C and G3460A mitochondrial DNA variants associated with Lеber’s Hereditary Optic Neuropathy (LHON).

The other affected brother (patient 2), aged 26 years, had had optic atrophy since childhood. He also had progressive myopia − 18.0D in both eyes. Perinatal and early psychomotor development were unremarkable. In childhood, he was noted to have learning difficulties and attention deficit disorder. There was no history of seizures or evidence of cognitive or neurological regression or deterioration. Corrected visual acuity at the time of this study was 20/150 in both eyes, with a normal anterior segment with no evidence of iron deposits (Fig. 2B, C) and prominent optic disc pallor (Fig. 2F, G). OCT revealed general thinning of the peripapillary nerve fibers (average thickness 46 μm bilaterally) (Fig. 2E). A 3-year-old girl from another family (patient 3) was referred due to global developmental delay starting at 6 months and followed by intractable seizures. Perinatal history was unremarkable. At the time of evaluation, she had no speech or communication and was wheelchair-dependent. Both parents are healthy; no relevant family history was reported.

Ophthalmological examination revealed pathologic myopia of 9.5D and 8.0D in the right and left eye, respectively, with fix and follow visual acuities. Normal anterior segments and bilateral temporal optic disc pallor with normal retinas were noted. Full-field ERG was normal in photopic conditions, ruling out extensive cons dysfunction. Scotopic testing showed a mildly reduced response, signifying mild rod dysfunction. MRI showed a thin corpus callosum and white matter volume depletion. Posterior segment imaging, including OCT scans and visual field testing, was not performed owing to the girl’s young age. A clinical comparison of the affected patients is presented in Table 1.

**Table 1:** Clinical comparison of affected patients

Feature	F1-II-1	F1-II-2	F2-II-1
Age	32	26	3
Sex	Male	Male	Female
Genotype	c.236-1G > T	c.236-1G > T	c.19 C > T / WT
Visual acuity	20/150 both eyes	20/150 both eyes	Fix and follow
Refractive error	-15.0 D	-18.0 D	-9.5 / -8.0 D
Optic nerve status	Atrophy, disc pallor	Atrophy, disc pallor	Temporal pallor
OCT	RNFL thinning	RNFL thinning	Not performed
ERG	Not reported	Not reported	Mild rod dysfunction
Visual field	Temporal defect	Not available	Not available
Neurological findings	Mild coordination issues, OCD, no regression	Learning difficulties, no regression	Global developmental delay, epilepsy
MRI	Optic nerve atrophy	Optic nerve atrophy	Thin corpus callosum
Brain iron on SWI	None detected	None detected	Not performed

Legend (Table 2):

D – Diopters;

OCT – Optical coherence tomography;

RNFL – Retinal nerve fiber layer;

ERG – Electroretinography;

SWI – Susceptibility-weighted imaging;

GDD – Global developmental delay

### Control group

The control group was partially reported in a previous study by our group and included 28 healthy volunteers, 21 females and 7 female, aged 18–50 years (mean 28.5 ± 9.09 years) [15]. All participants were regularly engaged in studio-based physical activity and were free of known systemic or ocular disease. None reported use of contact lenses, ophthalmic medications, or a history of dry eye symptoms, glaucoma, or anterior segment abnormalities. Tear sampling was performed under uniform conditions. This control group served as the baseline for comparison of tear trace element concentrations in the study patients. Genotype and clinical features of all study participants are summarized in Table 2.

**Table 2:** Genotype and clinical features of study subjects

Pedigree ID	Sex	Relationship	Genotype	Clinical Status
F1-I-1	F	Mother	c.236-1G > T / WT	Carrier, optic disc pallor
F1-I-2	M	Father	WT	Unaffected
F1-II-1	M	Son (Patient 1)	c.236-1G > T	Affected, optic atrophy
F1-II-2	M	Son (Patient 2)	c.236-1G > T	Affected, optic atrophy
F1-II-3	F	Daughter	WT/WT	Unaffected
F1-II-4	F	Daughter	c.236-1G > T / WT	Carrier, myopia
F1-II-5	M	Son	WT	Unaffected
F1-II-6	M	Son	WT	Unaffected
F2-I-1	M	Father	WT	Unaffected (parent of de novo case)
F2-I-2	F	Mother	WT / WT	Unaffected (parent of de novo case)
F2-II-1	F	Daughter (Patient 3)	c.19 C > T / WT	Affected, GDD, epilepsy, optic atrophy
Controls (*n* = 28)	M/F	Healthy volunteers	Not genotyped	No ocular or neurological findings; normal tear iron levels

Legend (Table 1):

F1-family 1, F2-family 2; F-female, M-male

GDD – Global developmental delay; WT – Wild-type allele

Not genotyped – Genetic testing was not performed on control participants

### Molecular genetics

**Fig. 4:**
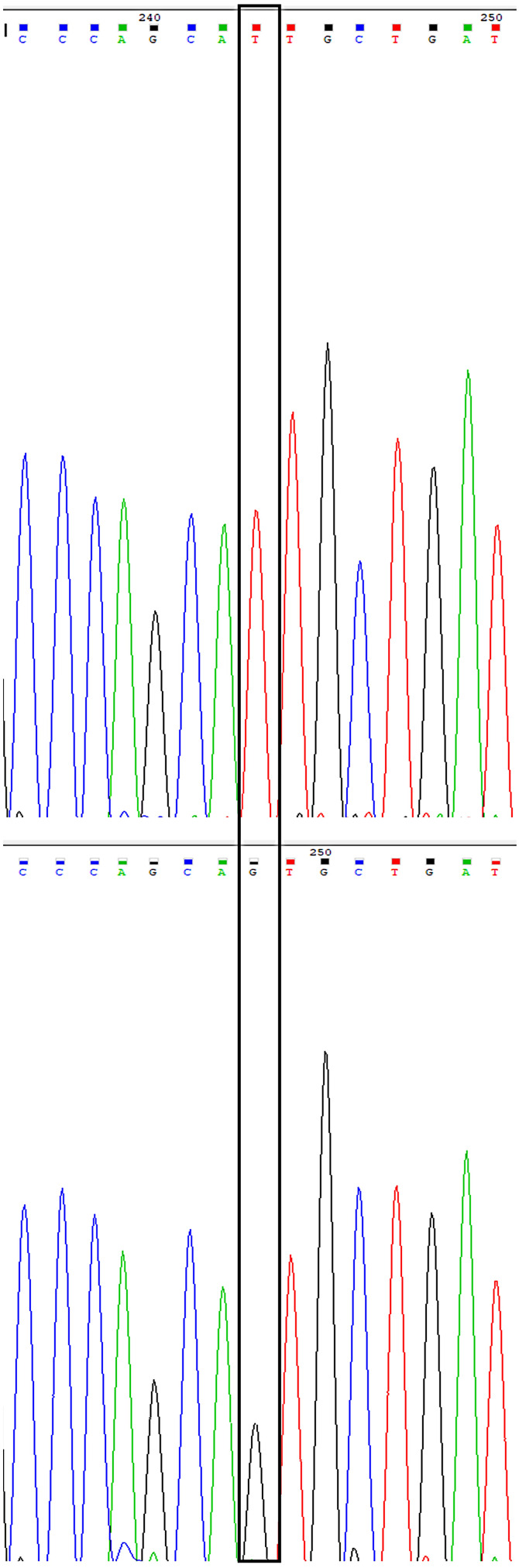
Sanger sequencing confirmation results of the *WDR45 (NM_001029896.2)*:c.236-1G > T variant. Sequencing results of the Hemizygous carrier patient presented in the upper electropherogram, the results of the healthy brother confirming wild type genotype are presented in the electropherogram below

Sanger sequencing performed on Patient 2, previously described by Gazit et al. [14], identified a hemizygous pathogenic splice-site variant in *WDR45 (NM*_001029896.2): c.236-1G > T. Segregation analysis by Sanger sequencing in the remaining family members demonstrated that both affected brothers were hemizygous for the variant, while the mother and one sister were heterozygous carriers. The two unaffected brothers and an additional sister did not carry the variant (Fig. 4). This variant was classified as pathogenic based on predicted splicing disruption, absence from population databases, segregation with the phenotype, and supportive transcriptomic data (ACMG criteria: PVS1, PM2, PS4, PP5). In patient 3, a heterozygous de novo nonsense variant in *WDR45*: c.19 C > T; p.Arg7Ter, previously identified by trio exome sequencing with her parents, was confirmed by Sanger sequencing. The variant was classified as pathogenic based on its truncating effect, confirmed de novo status, absence from population databases, and prior association with *WDR45*-related disorders (ACMG: PVS1, PS2, PM2).

### Targeted WDR45 RNA sequencing analysis

Targeted *WDR45* RNA sequencing analysis was performed to assess the expression levels of the *WDR45* in patient 2. At the c.236-1G > T location in *WDR45*, there is a redundancy at the exon/intron boundary for two different splice acceptor sites utilized by two different transcripts. On targeted RNA sequencing analysis performed in patient 2, the c.236-1G > T variant disrupted the splice acceptor site specific for the transcript NM_001029896.2, but not the splice acceptor site specific for the transcript NM_007075.3. The transcriptome data showed a decrease in transcripts utilizing the splice site for transcript NM_001029896.2, and a reciprocal increase in transcripts utilizing the splice site for transcript NM_ 007075.3 compared to tissue-matched controls. These findings are supportive but limited, as the analysis was performed on peripheral blood and does not reflect tissue-specific expression or function.

### PIXE analysis

**Fig. 5:**
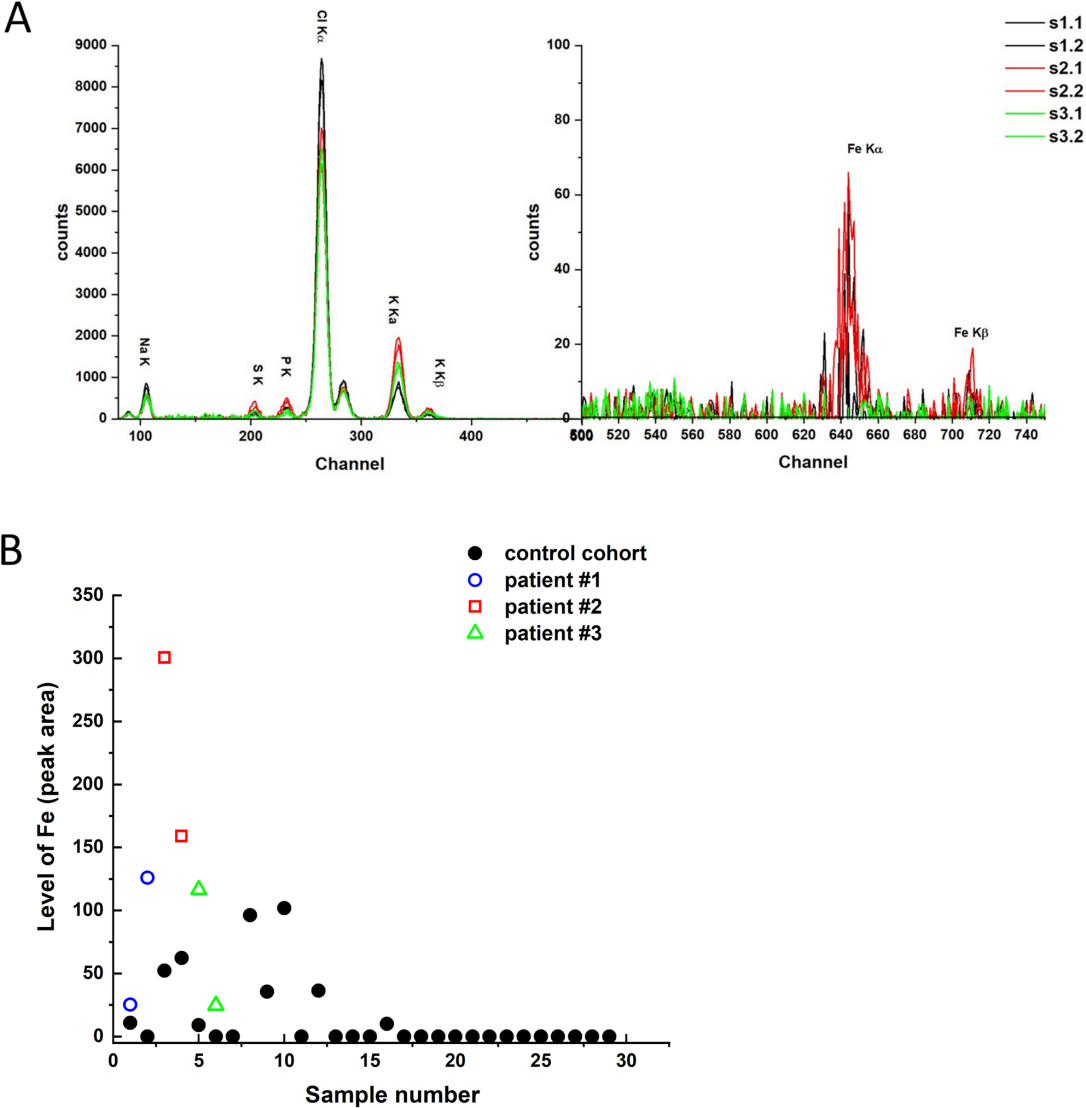
PIXE analysis of tear samples. (**A**) PIXE spectra were obtained from tear samples of three patients, with two samples analyzed per patient (one from each eye). Spectral data were processed using GUPIX software with a least-squares fitting approach. The evaluated peak area, particularly for the Fe K lines, was used to quantify iron levels in each sample. (**B**) Results were compared to tear samples from the control group. Each data point represents the average of three measurements taken from different areas of the Schirmer strip for each individual sample

The control cohort was partially described by our group previously, with tear iron levels averaging 14.8 peak area units [15]. All three patients with WDR45 X-linked optic atrophy exhibited elevated iron levels in their tears compared to healthy controls (Fig. 5A, B).

There were no differences from controls in tear levels of other trace elements. Serum iron levels in the siblings were within normal limits: 131 µg/dL in the first brother and 91 µg/dL in the second (reference range 65–176 µg/dL for adult males).

## Discussion

*WDR45* is an X-linked dominant gene, with more severe expression typically seen in females [1, 6]. We describe two brothers and one unrelated girl with optic atrophy associated with pathogenic variants in *WDR45*. All three developed high pathological myopia in early childhood. Tear iron levels were elevated in all cases, while brain MRI showed no iron accumulation despite the pathogenic genetic findings. In this study, we evaluated tear iron levels as a potential marker of optic atrophy and for their possible relevance to the underlying molecular mechanism.

WDR45 disorders have been classically associated with neurodegeneration and cognitive decline, particularly in females. In this study, the two affected brothers presented with optic atrophy and with early developmental and behavioral manifestations, but did not demonstrate any evidence of neurological or cognitive deterioration in adulthood. The girl’s more severe presentation may relate to a de novo nonsense variant causing complete loss of function, without compensation from random X-chromosome inactivation.

The role of autophagy has been explored in the context of iron control. In an experimental model, the induction of autophagy has been shown to influence the manifestation of neurological signs when autophagy inducers were used [19]. In syndromes like BPAN that present because of pathogenic variants in the *WDR45* gene, regression and iron accumulation in the basal ganglia are commonly noted [3, 4].

The optic nerve is highly dependent on uninterrupted mitochondrial energy production to support axonal function and survival. Mitochondria are also associated with various types of syndromes of neurodegeneration with brain iron accumulation [20–25]. This might cause early damage to the optic nerve. Other hereditary optic neuropathies also involve mitochondrial and autophagy-related pathways. For example, OPA1 plays a role in regulating mitochondrial fusion [26], while LHON is caused by defects involving the DNA of the mitochondria [27, 28].

*WDR45* has multiple splice transcripts, and their tissue distribution and function are still not fully defined. In our patients, the variant disrupted the splice acceptor site of NM_001029896.2, while NM_007075.3 remained intact. In peripheral blood RNA, the affected transcript was reduced, with a reciprocal increase in the intact transcript. This transcript imbalance could disturb tissue-specific iron regulation and may relate to the increased tear iron levels. Similar splice-related effects in other genes have been associated with autophagy dysfunction and iron deposition [29], and *WDR45* knockout models show axonal injury with impaired autophagy [30].

Both brothers had serum iron levels within the normal range, arguing against systemic iron overload. The elevated iron levels detected in tears may therefore reflect localized ocular dysregulation rather than circulating iron status. At present, the relationship between tear iron composition and systemic iron homeostasis remains poorly defined.

Previous work has demonstrated that PIXE can reliably quantify trace elements in small tear samples collected using Schirmer strips [17]. Using this validated approach, we identified elevated tear iron levels in all three affected patients. These findings suggest that tear analysis may complement ophthalmologic evaluation in the assessment of selected neurodegenerative conditions.

This study has several limitations. Magnetic resonance spectroscopy was not performed and could have provided additional information on tissue metabolism. Serum iron levels were not assessed in the control group, tear volume was not quantified, and X-chromosome inactivation or allele-specific expression was not evaluated in the affected female patient.

Despite these limitations, our findings are consistent with a role for WDR45 dysfunction in altered iron regulation, potentially through impaired ferritinophagy. The relationship between this process and the development of early, high myopia remains unclear and warrants further investigation. Although no direct mechanistic link between tear iron levels and brain iron accumulation has been established, the elevated tear iron observed in our patients suggests that local ocular iron dysregulation may occur independently of, or precede, detectable central nervous system involvement. Tear iron analysis may therefore represent a non-invasive exploratory biomarker in selected clinical contexts.

## Data Availability

The raw data that support the findings of this study are not openly available due to the need to protect study participant privacy and are available from the corresponding author upon reasonable request. Data are located in controlled access data storage at Bnai Zion Medical Center.
